# Astaxanthin Protects PC12 Cells against Homocysteine- and Glutamate-Induced Neurotoxicity

**DOI:** 10.3390/molecules25010214

**Published:** 2020-01-05

**Authors:** Chi-Huang Chang, Kuan-Chou Chen, Kuo-Chun Liaw, Chiung-Chi Peng, Robert Y. Peng

**Affiliations:** 1 Department of Biotechnology, College of Medical and Health Care, Hungkuang University, No. 1018, Sec. 6, Taiwan Boulevard, Shalu District, Taichung City 43302, Taiwan; ok1456@sunrise.hk.edu.tw (C.-H.C.); d002089009@tmu.edu.tw (K.-C.L.); 2 Department of Urology, School of Medicine, College of Medicine, Taipei Medical University, 250 Wu-Shing St., Taipei 11031, Taiwan; kuanchou@tmu.edu.tw; 3 Department of Urology, Taipei Medical University Shuang-Ho Hospital, 291, Zhong-Zheng Rd., Zhong-He, Taipei 23561, Taiwan; 4 Graduate Institute of Clinical Medicine, College of Medicine, Taipei Medical University, 250 Wu-Hsing Street, Taipei 11031, Taiwan

**Keywords:** PC12 cells, glutamate (Glu), homocysteine (Hcy), intrinsic apoptotic pathways, neuroprotective, astaxanthin (ATX)

## Abstract

Memory impairment has been shown to be associated with glutamate (Glu) excitotoxicity, homocysteine (Hcy) accumulation, and oxidative stress. We hypothesize that Glu and Hcy could damage neuronal cells, while astaxanthin (ATX) could be beneficial to alleviate the adverse effects. Using PC12 cell model, we showed that Glu and Hcy provoked a huge amount of reactive oxygen species (ROS) production, causing mitochondrial damage at EC_50_ 20 and 10 mm, respectively. The mechanisms of action include: (1) increasing calcium influx; (2) producing ROS; (3) initiating lipid peroxidation; (4) causing imbalance of the Bcl-2/Bax homeostasis; and (5) activating cascade of caspases involving caspases 12 and 3. Conclusively, the damages caused by Glu and Hcy to PC12 cells can be alleviated by the potent antioxidant ATX.

## 1. Introduction

Glutamate is the most well-known excitatory neurotransmitter, occurring in over 50% of nervous tissues [1]; it is involved in the process of learning, cognition, and neurodegeneration [2]. Two major classes of glutamate receptors are recognized, the ionotropic glutamate receptors (iGluRs) and the metabotropic glutamate receptors (mGluRs) [3]. All the iGluRs function as nonselective cation channels, allowing the passage of Na^+^, K^+^, and small amounts of Ca^2+^ in some cases [2]. The mGluRs modify neuronal and glial excitability through G protein subunits acting on membrane ion channels and second messengers such as diacylglycerol and cAMP [2]. Excess extracellular glutamate level could induce brain lesions, neuronal death, and other pathological changes in several organs associated with endocrine function; this pathway is called excitotoxicity, which was coined by Olney (1969) [4]. During the development process for Alzheimer’s disease (AD), excessive activated extracellular glutamate receptors prompt changes in ion channels and signaling systems [5,6,7]. The intracellular calcium produces an avalanche of reactive oxygen species (ROS), leading to mitochondria-mediated apoptotic process, in particular, during such a pathological condition [5,6,7]. ROS and the oxidative-stress-induced injuries lead to nuclear degradation in both neuronal and vascular systems, causing early loss of cellular membrane asymmetry that mitigates inflammation and vascular occlusion [8]. The mechanism has been reported to involve the Wnt pathway and the serine-threonine kinase Akt as well as the downstream substrates like GSK-3β, Bad, and Bcl-xL [8].

Homocysteine (Hcy), a neurotoxic amino acid, accumulates in several neurodegenerative disorders, including AD [9,10]. In AD patients, the blood plasma Hcy level has been found to rise above 20 μM [2]. Hcy acts as an agonist at the glutamate binding site of the *N*-methyl-D-aspartate receptor [11]. The PC12 cell line, originally isolated from a pheochromocytoma in the rat adrenal medulla, has become an in vitro model for studying numerous problems in neurobiology, neurochemistry [12,13], and Alzheimer’s and Parkinson’s diseases [14]. Like glutamate, Hcy induced calcium influx, depolarization of mitochondrial membrane potential, and changes of Bcl-2 and Bcl-xL in the PC12 cell model [15]. Accumulating studies of animal and cell culture models have clearly shown that the ability of neurons to regulate cellular Ca^2+^ levels and dynamics properly is compromised by both oxidative stress and impaired cellular energy metabolism [16], which features the common molecular pathological mechanisms raised by glutamate and homocysteine. Abnormalities in calcium regulation in astrocytes, oligodendrocytes, and microglia have been documented in studies of experimental models of AD [17]. Human studies suggest that Hcy plays a role in brain damage and in cognitive and memory decline. Numerous studies in recent years have implicated the role of Hcy as a cause of brain damage [18].

Astaxanthin (ATX) is a red-orange xanthophyll carotenoid, exhibiting a diversity of bioactivities, including antioxidant and peroxyl radical scavenging, anti-inflammation, cardiovascular health protection, anticancer, and antioxidant bioactivity [19]. ATX, with unique cell membrane actions and diverse biological activities, acts as a potential neuroprotective agent for neurological diseases [20].

We hypothesize that Glu and Hcy may cooperatively attack the glutamate receptor; whether ATX can effectively issue its protective effect against the neurotoxicity induced by cotreatment with these two ligands is still unclear. To verify this, we carried out this present experiment.

## 2. Results and Discussions

### 2.1. Cell Viability Affected by Astaxanthin, Homocysteine, and Glutamate

**Figure 1:**
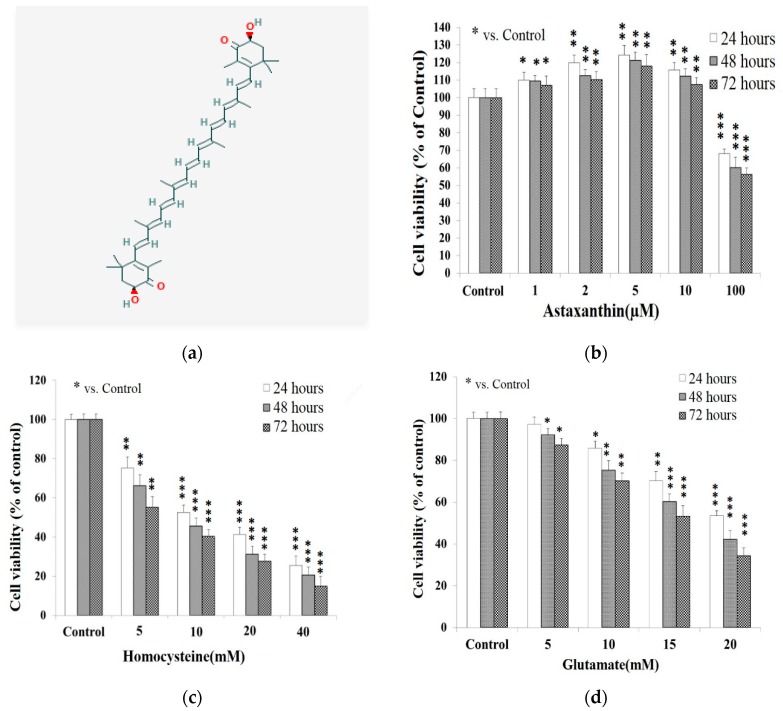
Chemical structure of astaxanthin and the effect of each compound of interest on the viability of PC12 cell line cultured for 24, 48, and 72 h. 24 h: empty bars; 48 h: gray bars; 72 h: dark net bars. PC12 cells were seeded onto a 24-well plate at 5 × 10^4^ cells/mL and cultured in serum-free medium overnight, then treated with astaxanthin (ATX), homocysteine (Hcy), or glutamate (Glu). (**a**) Chemical structure of ATX (depicted from PubChem, https://pubchem.ncbi.nlm.nih.gov/). (**b**) ATX (0–100 μM). (**c**) Hcy (0–40 mM). (**d**) Glu (0–20 mM). Data are expressed as means ± SD (*n* = 3). * *p* < 0.05; ** *p* < 0.01; *** *p* < 0.005 vs. control at the same time.

ATX (Figure 1a) did not show any cytotoxicity until up to a dose of 100 μM. Within the doses from 10 μM to 100 μM, it is evidently seen that the cell viability was inhibited in a dose-dependent manner (Figure 1b). The 50% inhibition only occurred at 100 μM after incubation for 72 h (Figure 1b), rather consistent with the observation of Ye et al. (2013) [21]. Both Hcy and Glu were revealed to be highly cytotoxic. The cytotoxicity also occurred in a dose-dependent manner. The IC_50_ was found to occur at 10 mM Hcy and 20 mM Glu at 24 h (Figure 1c,d). PC12 cells were found to be more susceptible to Hcy than Glu, with susceptibilities of 3.4%/mM and 1.3%/mM, respectively, at 48 h of incubation (Figure 1c,d). On the other hand, Hcy or Glu have been reported to induce apoptosis in neuronal cells by upregulating GRP78 [22,23,24].

A concentration of Hcy up to 10 μM has been measured in brain [25]. Elevated concentration of total Hcy in plasma (>12 μmol/L) is a risk factor for several diseases of the central nervous system [10]. However, more modest levels (15–50 mM) are found very commonly in the general population (a condition known as hyper-homocysteinemia) [26,27]. The cell viability could be affected by several factors including the kind of cell line and medium for incubation [28]. Froissard and Duval (1994) reported that glutamate (1–10 mM) led to a dose-dependent cell damage (70% of cell lysis at 10 mM) [29]. In contrast, according to Wang et al. (2016), 50% of severe suppression of Y79 cell viability by glutamate occurred at a dose of 20 mM [30]. Literature elsewhere also often used 25 mM of glutamate for conducting similar studies [5,31,32,33], consequently, a dose of 10 mM Hcy and 20 mM Glu has been adopted in this article.

### 2.2. Potentiation of Glutamate on the Cytotoxicity of Homocysteine

**Figure 2:**
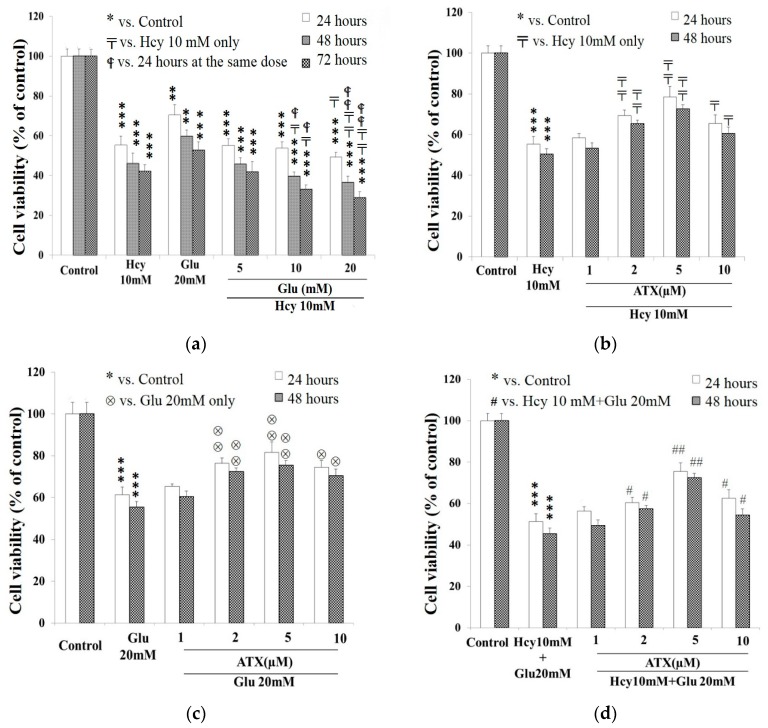
Combined effect of target compounds on the viability of PC12 cell line. PC12 cells were seeded onto 24-well plates at 5 × 10^4^ cells/mL and cultured in serum-free medium overnight, then treated with different combinations of ATX, Hcy and/or Glu separately. In all panels, empty bars: 24 h. In Figure 2a, gray bars: 48 h; dark net bars: 72 h. In Figure 2b–d, net dark bars: 48 h. (**a**) Glu (5–20 mM) plus Hcy (10 mM). (**b**) ATX (1–10 μM) plus Hcy (10 mM). (**c**) ATX (1–10 μM) plus Glu (20 mM). (**d**) ATX (1–10 μM) plus Hcy (10 mM) plus Glu (20 mM). Data are expressed as means ± SD (*n* = 3). *: compared to the control; 〒: vs. Hcy 10 mM at the same time; 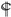: vs. 24 h at the same dose; ⊗: vs. Glu 20 mM at the same time; #: vs. Hcy 10 mM + Glu 20 mM at the same time. The significance of the difference was judged by confidence levels of * *p* < 0.05; ^#^ *p* < 0.05; **^〒^** *p* < 0.05; ^⊗^ *p* < 0.05; ^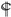 ^*p* < 0.05; ** *p* < 0.01; ^##^ *p* < 0.01; ^⊗⊗^ *p* < 0.01; ^
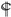

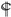
 ^*p* < 0.01; *** *p* < 0.005.

Both Glu and Hcy are cytotoxic, and we questioned whether these two compounds may exert any additive or synergistic effect on the cell viability. Glu at 5 mM seemed to be totally ineffective to potentiate the cytotoxicity of 10 mM Hcy. At a dose >10 mM (up to 20 mM), Glu significantly potentiated the Hcy cytotoxicity in a dose- and time-dependent manner (Figure 2a). Much of literature has evidenced that Hcy not only induces direct neurotoxicity, but also potentiates both amyloid-β and glutamate neurotoxicity [34]. Similarly, it has been revealed that activation of group III metabotropic glutamate receptors stimulates the excitotoxic action of Hcy and homocysteic acid [2]. Previously, Lecleric et al. evidenced the occurrence of NMDA receptor in PC12 cells and concluded that PC12 cells express predominantly the splice variant NMDAR1-4a and smaller amounts of NMDAR1-1a, NMDAR1-2a, and NMDAR1-3a [35]. More recently, Sibarov et al. implicated that GluN2A subunit-containing NMDA receptors as the preferential neural targets of Hcy [36]. Moreover, Hcy has been confirmed not only to be Ca^2+^- and NMDA receptor-dependent, but also Ca^2+^-independent, mainly mediated by the “synaptic type” GluN1/2 NMDAR [36]. Suggestively, the degree of binding by different ligands like Glu and Hcy for these receptor subunits and the outcome responses may be synergistically additive on one hand, but competitively expelling on the other hand.

### 2.3. Protective Effect of Astaxanthin against The Insult Exerted by Homocysteine and Glutamate

ATX at 2–5 μM significantly alleviated the cell viability from the insult caused by Hcy (10 mM) (Figure 2b), Glu (20 mM) (Figure 2c), and the combined Hcy (10 mM) plus Glu (20 mM) (Figure 2d) at 24 and 48 h. As seen, ATX at 2 and 5 μM secured the cell viability for about 12%–14% and 21%–22%, respectively, (Figure 2b) when insulted by Hcy. ATX at 2 and 5 μM secured the cell viability for about and 14%–19% and 19%–22% (Figure 2c) when insulted by Glu for 24–48 h, respectively. The alleviation against the combined insults was seen as rather comparable at 2 μM of ATX (11% and 13% for 24 and 48 h, respectively) but was much better at 5 μM ATX (26%) at 48 h (Figure 2d).

Literature has implicated that ATX inhibits homocysteine-induced neurotoxicity via alleviating mitochondrial dysfunction and signal crosstalk [37]. A similar result was found in in vitro cardiac cell cultures [38]. Previously, Zhang et al. found that ATX exerted neuroprotective effect via multiple signaling pathways including NFκB and MAPK pathways, and implicated ATX to be used as a prophylactic or remediation agent against neuronal disorder [39].

### 2.4. Effect of Astaxanthin on the Intracellular Calcium Ion Level Affected by Homocysteine and Glutamate

**Figure 3:**
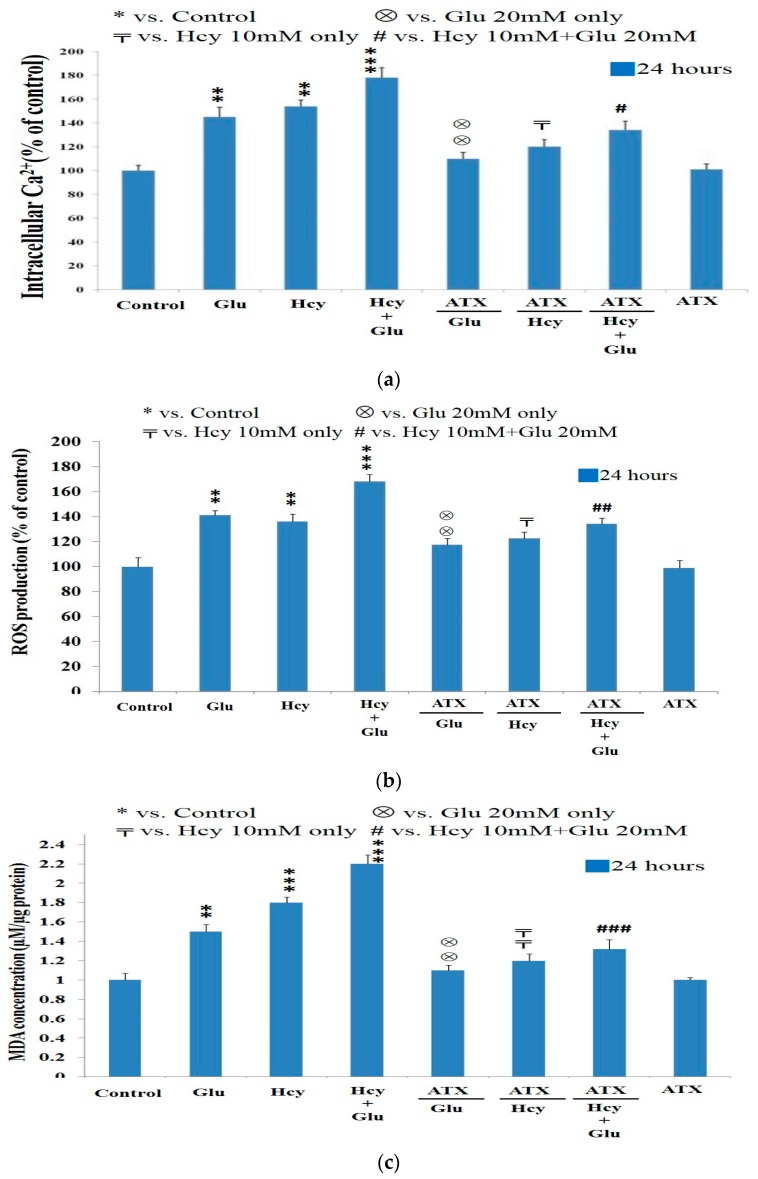
Protective effect of astaxanthin against the calcium ion influx and oxidative stress induced by target compounds. PC12 cells were cultured in serum-free medium overnight, then treated with different combinations of ATX (5 μM), Hcy (10 mM), and/or Glu (20 mM). (**a**) Intracellular calcium ion level. (**b**) Reactive oxygen species (ROS) production. (**c**) Malondialdehyde (MDA) production. Data are expressed as means ± SD (*n* = 3). *: compared to the control; ⊗: vs. Glu 20 mM only; 〒: vs. Hcy 10 mM only; #: vs. Hcy 10 mM + Glu 20 mM. The significance of the difference was judged by confidence levels of * *p* < 0.05; ^⊗^ *p* < 0.05; ^〒^ *p* < 0.05; ^#^ *p* < 0.05; ** *p* < 0.01; ^⊗⊗^ *p* < 0.01; ^##^ *p* < 0.01;^〒〒^ *p* < 0.01; *** *p* < 0.005; ^###^ *p* < 0.005.

Calcium ion dysregulation is relevant to the initiation of Alzheimer’s disease (AD), the PC12 cell model in reality has pertinently reflected such a possibility [40]. The calcium ion influx was highly raised by the insult of 20 mM Glu (144%), and/or 10 mM Hcy (153%), and/or the combined treatment (176%) (Figure 3a). ATX at 5 μM fully alleviated the calcium influx raised by 20 mM Glu, but was slightly less effective against that by Hcy (10 mM), and was much less effective against the combined therapy (133%) (Figure 3a), implicating insufficient mole number for the interactions between ATX and (Glu + Hcy); suggestively, a higher dose of ATX may be required for such a combined insult.

In addition, Hcy has been implicated to indirectly increase intracellular calcium levels by activating ionotropic and metabotropic receptors [41], which, compared with glutamate, may create the reasons to elicit different outcomes by such a combination of pharmacological actions of Hcy [2,36,41].

Two subcellular organelles, namely the mitochondria and endoplasmic reticulum (ER), are involved in the cellular pathogenesis of AD; an increased oxidative stress and dysregulation of calcium homeostasis also have been reported [42]. Owing to the important role of ER in the regulation of Ca^2+^-signaling, ER-mitochondrial distance in the neurons is tightly controlled in the physiological conditions. When the distance is decreased, Ca^2+^-overload occurs both in the cytosol and mitochondria [40]. The reduction in the distance between ER and mitochondria may be implicated in Alzheimer’s disease (AD) pathology by enhanced Ca^2+^-signaling [40]. Lin et al.’s study indicated that ATX attenuated glutamate-induced elevation of CHOP and ER chaperone glucose-regulated protein (GRP78), inhibiting glutamate-induced apoptosis through rescuing the redox balance and inhibiting glutamate-induced calcium influx [24].

### 2.5. Effect of Astaxanthin on the ROS Production Caused by Homocysteine and Glutamate

The level of reactive oxygen species (ROS) was highly induced by treating with Glu (20 mM), Hcy (10 mM), and the combined treatment for 24 h (Figure 3b). The ROS produced were effectively suppressed by ATX at 5 μM (Figure 3b).

The mitochondrial Ca^2+^-overload can lead to increased generation of reactive oxygen species, inducing the opening of the mitochondrial permeability transition pore and ultimately causing neuronal apoptotic and necrotic cell death [40]. In addition, the NO pathway relating with hyperexcitability is induced by homocysteine thiolactone (HcyT) [43]. ATX is a nutrient with unique cell membrane actions and diverse clinical benefits [44]. ATX is the most effective antioxidant. This molecule neutralizes free radicals or other oxidants by either accepting or donating electrons, without being destroyed or becoming a pro-oxidant in the process [44]. ATX blocks oxidative DNA damage and lowers C-reactive protein (CRP) and other inflammation biomarkers [44]. ATX-mediated neuroprotection in experimental models of neurological disorders involves antioxidant, anti-inflammatory, and antiapoptotic mechanisms [45,46,47].

### 2.6. Effect of Astaxanthin on the MDA Production Resulting from Insult of Homocysteine and Glutamate

MDA was highly stimulated when treated with Glu (20 mM), Hcy (10 mM), and the combined therapy to levels of 1.48, 1.80, and 2.20 μmol/μg of protein, respectively, which were suppressed by treatment with 5 μM ATX (Figure 3c), similar to the outcomes for Ca^2+^ influx (Figure 3a) and ROS induction (Figure 3b). ATX was evidenced to be a strong peroxyl radical scavenger, exerting a strong protective effect on the human brain [19,48].

### 2.7. Western Blot Analysis Indicated Astaxanthin Restored Bax and Bcl-2 Homeostasis

Bcl-2 family members either promote or repress programmed cell death. Bax, a proapoptotic member of the Bcl-2 family of proteins, is a pore-forming, mitochondria-associated protein [49] as well as an activator for the mitochondrial permeability transition pore (mPTP) [50].

**Figure 4:**
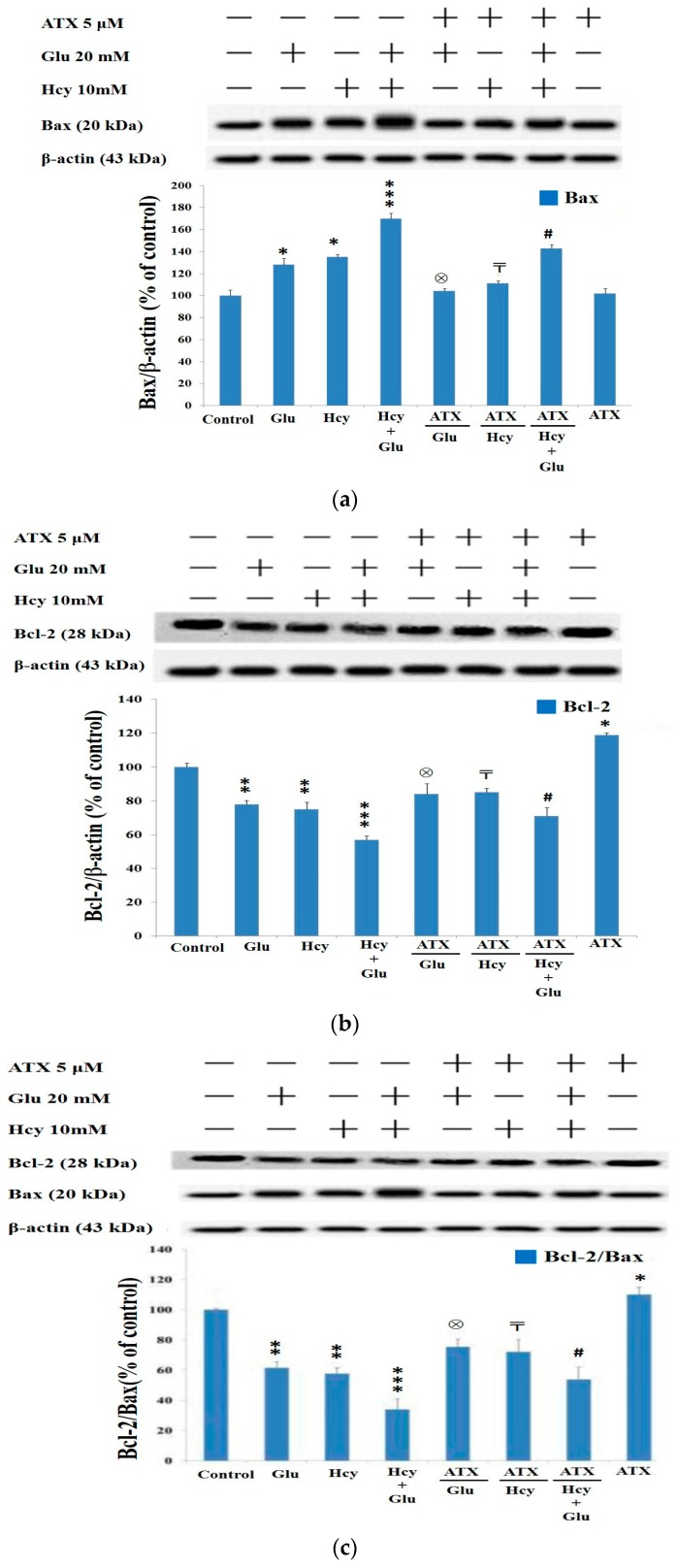
Western blot and quantified protein expression affected by different treatments. (**a**) Bax; (**b**) Bcl-2; and (**c**) Bcl-2/Bax. Western blot analysis quantified into bar diagram. Data are expressed as means ± SD (*n* = 3). *: vs. control; ⊗: vs. Glu 20 mM only; 〒: vs. Hcy 10 mM only; #: vs. Hcy 10 mM + Glu 20 mM. The significance of the difference was judged by confidence levels of * *p* < 0.05; ^⊗^ *p* < 0.05; ^〒^ *p* < 0.05; ^#^ *p* < 0.05; ** *p* < 0.01; *** *p* < 0.005.

Western analysis revealed that the Bax (proapoptotic protein) level was highly upregulated to 128%, 134%, and 170% by Glu (20 mM), Hcy (10 mM), and the combined treatments, respectively (Figure 4a). Interestingly, results clearly implicated the synergistic effect of Glu plus Hcy, i.e., 70% > (28% + 34%). ATX at 5 μM completely attenuated the suppression caused by glutamate or homocysteine alone and also ameliorated significantly that caused by the combined treatments of glutamate with homocysteine (Figure 4a). Conversely, Bcl-2 was severely down-regulated by the insult of Glu (20 mM), Hcy (10 mM), and the combined treatment to 78%, 76%, and 56%, respectively (Figure 4b). Hcy induced neurotoxicity via causing mitochondrial dysfunction to regulate Bcl-2 family and opening of mitochondrial permeability transition pores [37]. Our results have revealed that ATX was unable to have completely attenuated such inhibitions. Although ATX at 5 μM alone was able to increase the Bcl-2 level (antiapoptotic protein) to 116%, compared to 100% of the control (Figure 4b), the % recovery only attained 83% and 84% by the single insult exerted by Glu and homocysteine, respectively, and 70% by the combined insults. As the counterbalance of Bcl-2 and Bax is responsible for the homeostasis of the intrinsic pathway, we further examined the variation of Bcl-2/Bax ratio (Figure 4c). More apparent results were seen when insulted by Glu, Hcy and the combined therapy; the Bcl-2/Bax ratios reached 60%, 58%, and 34%, respectively, compared with 100% of the control (Figure 4c), for which ATX at 5 μM was found to have attenuated the levels to only 75%, 72%, and 52%, respectively, only. ATX (5 μM) alone increased the ratio to 108% (Figure 4c). In contrast, Wang et al.’s study showed the complete reversal of Hcy (8 mM)-induced neurotoxicity after treatment with ATX (5 μM) [37]; suggestively, such a deviation could be due to the difference in the cell model and dose of Hcy used.

In cultured cells, astaxanthin protected the mitochondria against endogenous oxygen radicals, conserved their redox (antioxidant) capacity, and enhanced their energy production efficiency [44]. Two distinct mechanisms leading to cytochrome C release were described by Eskes et al. (1998): one being stimulated by calcium and inhibited by cyclosporine A; the other being Bax-dependent, Mg^2+^ sensitive, but cyclosporine insensitive [49]. Apparently, the treatment of PC12 cells with Glu, Hcy and/or the combined therapy could suppress the cell viability (Figure 1) by up-regulating apoptosis (Figure 4) [51].

### 2.8. Effect of Astaxanthin on the Expression of Caspase-12 and Caspase-3 in PC12 Cells Treated with Homocysteine and Glutamate

**Figure 5:**
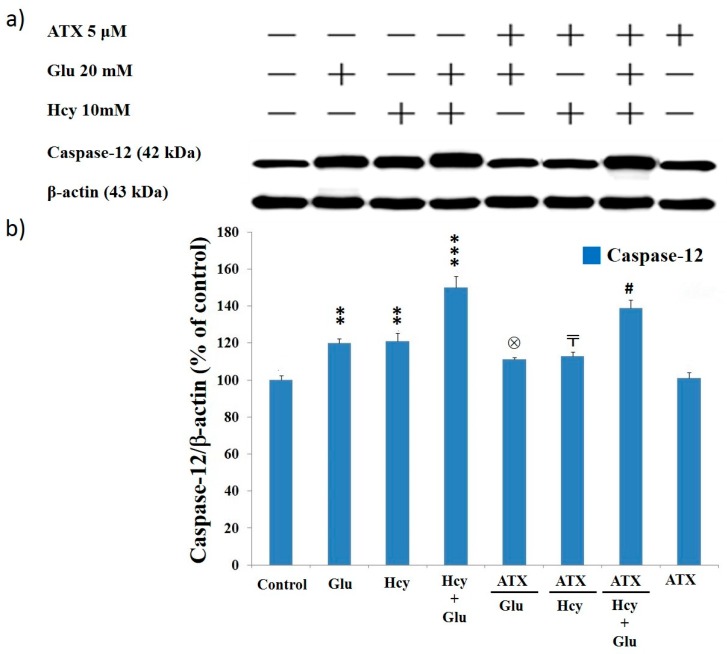
Caspase-12 expression affected by different treatments. (**a**) Western blot analysis; (**b**) quantified bar diagram. Data are expressed as means ± SD (*n* = 3). *: vs. control; ⊗: vs. Glu 20 mM only; 〒: vs. Hcy 10 mM only; #: vs. Hcy10 mM + Glu 20 mM. The significance of the difference was judged by confidence levels of * *p* < 0.05; ^⊗^ *p* < 0.05; ^〒^ *p* < 0.05; ^#^ *p* < 0.05; ** *p* < 0.01; *** *p* < 0.005.

**Figure 6:**
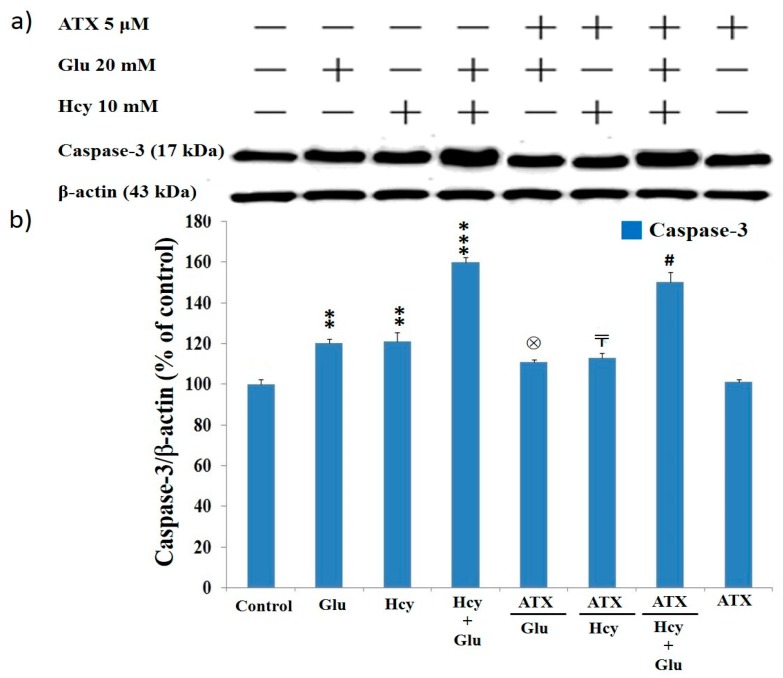
Caspase-3 expression affected by different treatments. (**a**) Western blot analysis; (**b**) quantified bar diagram. Data are expressed as means ± SD (*n* = 3). *: vs. control; ⊗: vs Glu 20 mM only; 〒: vs. Hcy 10 mM only; #: vs. Hcy10 mM + Glu 20 mM, The significance of the difference was judged by confidence levels of * *p* < 0.05; ^⊗^ *p* < 0.05; ^〒^ *p* < 0.05; ^#^ *p* < 0.05; ** *p* < 0.01; *** *p* < 0.005.

Results from Western blot analysis showed the levels of caspase-12 (42 kDa) and caspase-3 (17 kDa) were highly upregulated due to the insult of Glu (20 mM), Hcy (10 mM), and the combined treatment (Figure 5 and Figure 6). This again confirms that Glu, Hcy and/or the combined insult could suppress the cell viability (Figure 1), up-regulating apoptosis (Figure 4) and the activities of caspase-12 (Figure 5) and caspase-3 (Figure 6) and indicating the neurotoxic role of glutamate and Hcy to PC12 cells [51].

The level of caspase-12 was stimulated to 120%, 121%, and 148% due to the insult of Glu, Hcy, and combined treatment, respectively, comparing to 100% of the control (Figure 5). ATX at 5 μM was able to almost completely alleviate the adverse effects caused by Glu and Hcy (105% and 106%) but was only slightly effective for the combined treatment (138%) (Figure 5).

A similar trend was seen for caspase-3 (Figure 6). Glu (20 mM), Hcy (10 mM), and the combined therapy upregulated the level of caspase-3 to 119%, 120%, and 158%, respectively, compared to 100% of the control (Figure 6). The ameliorating effect of ATX (5 μM) was merely moderate. The percent suppression reached only 110%, 110%, and 148% for Glu (20 mM), Hcy (10 mM), and the combined therapy, respectively (Figure 6).

Caspase-12, expressed in mouse and human, is classified as an inflammatory caspase [52] that mediates ER-specific apoptosis pathway and contributes to Aβ neurotoxicity [52,53]. Moreover, is was found to mediate carbon tetrachloride-induced hepatocyte apoptosis in mice through the activation of the downstream effector caspase-3 directly and/or indirectly via caspase-9 activation [54]. Caspase-12 inhibition reduced stretch-induced apoptosis, and caspase-12 activated caspase-3 to induce apoptosis. Thus, caspase-12 plays an important role in stretch-induced apoptosis that is associated to endoplasmic reticulum stress by activating caspase-3 [55].

The cytosolic Ca^2+^-overload (Figure 3a) can (1) hyperactivate Ca^2+^-dependent enzymes, which in turn regulate activities of proapoptotic Bcl-2 family proteins (Figure 4), causing mitochondrial outer membrane permeabilization and thereby resulting in the release of cytochrome c to activate caspase-3 (the intrinsic pathway) (Figure 6) [40]; (2) indirectly activate caspase-3 through the activation of caspase-12 (the ER-pathway) (Figure 5 and Figure 6) [40]; and (3) promote the production and aggregation of β-amyloid. The three pathways eventually trigger neuronal apoptotic cell death [40].

In summary, both Hcy and Glu provoked the intrinsic pathways, resulting in cell apoptosis. In the intrinsic pathway, ROS and calcium ion influx triggered imbalanced Bcl-2/Bax homeostasis and the release of cytochrome c, which in turn upregulated caspase-3 and provoked cell apoptosis. The role of ATX was seen to have dose-dependently alleviated all these adverse effects. Literature elsewhere has indicated that Aβ induced GRP78/Bip expression and activated caspases 4 and 12 [56], implicating the possible alternate target of ATX.

## 3. Materials and Methods

### 3.1. Chemicals

Astaxanthin (ATX) and other chemicals used in this experiment were purchased from Sigma Aldrich Co. (St. Louis, MO, USA) unless otherwise stated. RPMI-1640 medium, trypsin, Fungizone (C_47_H_73_NO_17_), L-glutamine, penicillin and streptomycin were purchased from Invitrogen (Thermo-Fischer Scientific, Waltham, MA, USA). Purified NGF was purchased from Sigma-Aldrich (Sydney, Australia).

### 3.2. Source of Cell Line

The cell line PC12 cells (BCRC 6008), originating from the pheochromocytoma on the rat adrenal gland, was purchased from the Bioresource Collection and Research Center (Hsin-Chu, Taiwan).

### 3.3. Cultivation of PC12 Cell Line

The method for cultivation of PC12 cell line was conducted as previously reported [14].

### 3.4. Preparation of Complete Medium

RPMI 1640 medium was prepared according to the supplier’s protocol in reverse osmosis/double distilled water (https://biocyclopedia.com/index/cellbiology_methods/cultured_for_neuronal_pc12_cells.php) [57].

### 3.5. Preparation of Differentiating Medium

Serum-free RPMI 1640 medium stored at 4 °C was used. The PC12 cells were dislodged from stock culture dishes and triturated well using a glass Pasteur pipette to break up cell clumps. The cells were seeded onto 150-mm, 100-mm and 35-mm-dishes at densities of 5 × 10^6^, (1–2) × 10^6^, and (2–5) × 10^6^ cells, respectively, on poly-l-lysine-coated dishes and cultured in medium supplemented with a final concentration of NGF at 50 ng/mL. The cells were incubated at 37 °C under saturated water vapor and 7.5% CO_2_ atmosphere. The medium was changed three times per week. The culture was observed for 72 h. According to the instruction, by 7–10 days of treatment, at least 90% of the cells can generate neurites. (https://biocyclopedia.com/index/cell_biology_methods/cultured_for_neuroanal_pc12_cells.php) [57]. The differentiated cells were roughly counted by a hematocytometer.

### 3.6. Preparation of Astaxanthin Solution

The work of Bolin et al. (2010) was followed for preparation of astaxanthin DMSO stock solution, which was stored at −20 °C in a brown-colored Eppendorf tube. Appropriate dilution was performed before use [58].

### 3.7. Preparation of Homocysteine and Glutamate Solutions

The work of Zhou et al. (2005) was followed to prepare homocysteine DMSO solution [59], while the method of Kawakami et al. (2009) was conducted to prepare glutamic acid solution (pH 7.0) (denoted as Glu) [60]. With a slight modification, an appropriate amount of Hcy was weighed, dissolved in DMSO, and stored at −20 °C in a brown-colored Eppendorf tube. Appropriate dilution was performed before use.

### 3.8. MTT Assay Cell Viability Test

The cell viability test with MTT assay was carried out as previous reported [14]. The doses of Glu and Hcy were applied as indicated. The absorbance was read with ELISA Reader (ClarioStar, BMG Labtech Japan Ltd., Saitama, Japan) at 570 nm. Cell viability was calculated according to Equation (1):

Cell viability = (*A*s/*A*c) × 100%, (1)

where *A*s is the absorbance of the sample and *A*c is the absorbance of the control.

### 3.9. The Cytotoxicity of Astaxanthin

Test for ATX cytotoxicity was conducted as previously reported [45]. The dose of ATX was applied to 24-well culture plates as indicated. The cell viability was calculated according to Equation (1).

### 3.10. Protective Effect of Astaxanthin against the Cytotoxicity of Homocysteine, Glutamate, or Homocysteine Plus Glutamate

The PC12 cells were seeded at a density of 7 × 10^4^ cells/well and incubated for 24 h until adhesion. The medium was changed to the differentiating media containing ATX at 0.0, 1.0, 2.0, 5.0, 10.0, and 100.0 μM and incubated for 24 h The medium was replaced fresh with differentiating medium. Hcy (10 mM), Glu (20 mM), or Hcy (10 mM) plus Glu (20 mM) were added and the incubation was continued for 24 and 48 h. MTT assay was carried out as mentioned above. The cell viability was calculated according to Equation (1).

### 3.11. Determination of Intracellular Calcium Ion Concentration

The work of Sul et al. (2009) was followed to determine the intracellular calcium ion concentration [61]. In brief, PC12 cells were seeded onto 24-well plates at a density 7 × 10^4^ cells/well and incubated for 24 h until entirely adhered. The following protocol was carried out as cited. The doses of ATX, Hcy, and Glu were 5 μM, 10 mM, and 20 mM respectively. The final supernatant was discarded, and the pellets were rinsed with PBS. Then, 1× trypsin was added and incubated at 37 °C for 5 min. The cell density was counted with the hematocytometer, the cells were seeded onto 96-well plates at a density 2 × 10^4^ cells/well, and the medium was replaced fresh with the incomplete medium containing 1 μM Fluo-3/AM (C_51_H_50_C_l2_N_2_O_23_) and incubated at 37 °C for 30 min avoiding direct sunlight. The fluorescence produced after the reaction of Fluo-3/AM with calcium ions was measured at excitation wavelength *E*_x_ = 488 nm, and emitted wavelength *E*_m_ = 532 nm. The untreated sample was used as the blank and set at 100% to estimate the change of intracellular calcium ion concentration within different groups.

### 3.12. Determination of Intracellular Reactive Oxygen Species (ROS)

According to Zhang et al. (2008), the intracellular ROS level was determined [62]. The cultivation of PC12 cells and the doses of ATX, Hcy, and Glu were similarly conducted as mentioned in the above section. The final culture was centrifuged at 12,500× rpm for 20 min. The supernatant was discarded, and the pellets were rinsed with PBS. Then, 1× trypsin was added and incubated at 37 °C for 5 min. The cell density was counted with the hematocytometer, the cells were seeded onto 96-well plates at a density 2 × 10^4^ cells/well, and the medium was replaced fresh with the incomplete medium containing 10 μM 2′,7′-dichlorodihydrofluorescein diacetate (H_2_DCFDA) and incubated at 37°C for 30 min avoiding direct sunlight. The fluorescence of the product dichlorofluorescein (DCF) produced after the reaction of H_2_DCFDA with ROS was measured at excitation wavelength *E*_excitation_ = 488 nm, and emitted wavelength *E*_emission_ = 532 nm using a fluorescence ELISA Reader (Bio-Rad, Hercules, CA, USA). The untreated sample was used as the blank and set at 100% to calculate the amount of ROS produced within different groups.

### 3.13. Protein Extraction

Method of protein extraction was carried out as previously cited [63]. The cultivation of PC12 cells and the doses of ATX (5 μM), Hcy (10 mM), and Glu (20 mM) were similarly conducted as mentioned in the above section. The protein content of the final supernatant was determined using the authentic bovine serum protein to establish the calibration curve, and the results were expressed in μg/μL. The remaining supernatant was transferred to a new 5-mL Eppendorf tube and stored at –80 °C in a freezer to keep the integrity of the cells for further use.

### 3.14. Assay for the Malondialdehyde (MDA) Concentration

The determination of MDA was carried out using the protocol reported by Chang et al. (2011) [64]. The final OD was read at 532 nm using an ELISA Reader (Bio-Rad, CA, USA). Authentic 1,1,3,3-tetramethoxy propane (TMP) was used to establish the calibration curve, from which the concentration of MDA in samples was calculated and expressed in μM/μg protein.

### 3.15. Western Blot Analysis

The SDS PAGE (7.5%–12%) electrophoresis was conducted at 100 V as instructed by the manufacturer (Life Science, CA, USA). The 100 μg of sample protein was loaded in each well. The primary antibodies of monoclonal anti-βactin, polyclonal anti-Bax, polyclonal anti-cleaved caspase-3 (Cell Signaling Technology, Danvers, MA, USA), polyclonal anti-cleaved caspase-12, and polyclonal anti-BcL-2 (BioVision Inc., Milpitas, CA, USA) were applied as 1/1000 of dilution and left in a 4 °C ice box overnight. The PVDF membranes were rinsed with TBST buffer once for 30 min. The secondary antibodies Gt × Ms IgG(H + L) HRP and goat anti-rabbit IgG pAb HRP (Stressgen Biotechnologies Corporation Corporate, San Diego, CA, USA) were applied as 1/5000 of dilution and left in 4 °C ice box for 30 min. while shaken. The membranes were rinsed with TBST buffer twice, each time for 30 min. Enhanced chemiluminescence (ECL) was added and mixed well for 5 min to facilitate the reaction. The emitted chemiluminescence was taken by the Hansor LIS02 photo system, and the intensity was quantified with Image J (NIH Image, Bethesda, MD, USA).

### 3.16. Statistical Analysis

Data obtained in the same group were analyzed using the SPSS 10.0 statistical software (SPSS, Chicago, IL, USA). Analysis of variance (ANOVA) and Tukey’s test were used to analyze the variance. Data are expressed as means ± SD from triplicate experiments. Data with significant differences between groups were identified with statistical level of *^,^^⊗,#,^^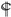,^**^〒^** *p <* 0.05; ^**,^^⊗⊗,##,^^
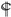

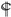
,^**^〒〒^** *p* < 0.01; *** ^,^^⊗⊗⊗,###,^^
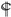

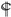

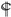
,^**^〒〒〒^** *p* < 0.005. 

## 4. Conclusions

**Figure 7:**
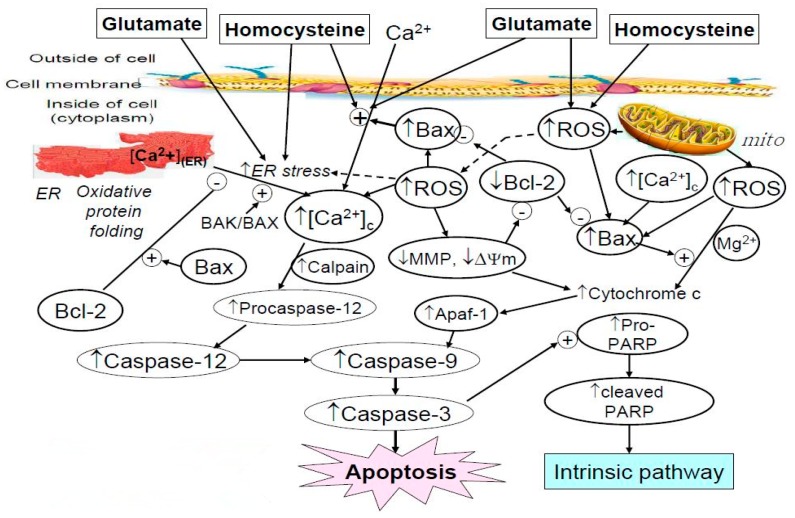
Summary of the apoptotic pathways induced by glutamate and homocysteine. This figure shows how glutamate and homocysteine induce mitochondrial damages, while astaxanthin alleviates the upregulation of Ca^2+^ influx, ROS, Bax, caspase-12, and caspase-3 and the downregulation of Bcl-2. ER: endoplasmic reticulum; mito: mitochondria; ROS: reactive oxygen species; MMP and Ψ_m_: mitochondrial membrane potential. Symbols expressed in larger characters are the experimental results, while symbols in smaller characters are pathway-linking items. PARP: Poly(ADP-ribose) polymerase; Apaf-1: (apoptotic protease activating factor 1).

Glutamate and homocysteine can cooperatively, but not additively or synergistically, damage neuronal cells as evidenced in the PC12 cell model. Also, homocysteine and glutamate compromise neuronal homeostasis by multiple and divergent routes. Both of them can provoke cell apoptosis via oxidative stress and the intrinsic pathway. The mechanisms of action may involve (1) increasing calcium influx; (2) producing ROS; (3) initiating lipid peroxidation (as evidenced by the huge production of MDA); (4) causing imbalance of the Bcl-2/Bax homeostasis; and (5) activating a cascade of caspases involving caspase-12 and caspase-3 (Figure 7). Meanwhile, ATX can protect the neuron-mimic PC12 cell viability via alleviating these adverse effects. Suggestively, drugs that exhibit potent antioxidative capability can be considered beneficial for treatment of certain neuro-excitatory diseases.
